# Exploring levels of empathy and assertiveness in final year physiotherapy students during clinical placements

**DOI:** 10.1038/s41598-024-64148-8

**Published:** 2024-06-10

**Authors:** Juan-Elicio Hernández-Xumet, Alfonso‐Miguel García‐Hernández, Jerónimo‐Pedro Fernández‐González, Cristo-Manuel Marrero-González

**Affiliations:** 1 Movement and Health Research Group, Departamento de Medicina Física y Farmacología, Facultad de Ciencias de La Salud, Universidad de La Laguna (ULL), La Laguna, Spain; 2 Hospital Universitario Nuestra Señora de Candelaria, Servicio Canario de La Salud, Santa Cruz de Tenerife, Spain; 3 Departamento de Enfermería, Facultad de Ciencias de La Salud, Universidad de La Laguna (ULL), La Laguna, Spain; 4 Gerencia de Atención Primaria de Tenerife, Servicio Canario de La Salud, Santa Cruz de Tenerife, Spain

**Keywords:** Assertiveness, Empathy, Health occupations students, Learning, Physical therapy, Health care, Health occupations

## Abstract

Empathy and assertiveness are two essential soft skills for any healthcare professional's competence and ethical development. It has been shown that empathy can be influenced throughout the training of a future healthcare professional, particularly during the clinical placement period. This research aims to assess fourth-year physiotherapy students' empathic and assertive development before and after clinical placement. A longitudinal observational study was conducted with fourth-year physiotherapy students during the academic year 2022/2023. A preliminary assessment of empathy and assertiveness levels was carried out before the start of the clinical placement and at the end of the placement using the Individual Reactivity Index to assess empathy and the Rathus Test to assess assertiveness. The results show a statistically significant difference (*p* ≤ 0.05) in both the empathy subscales of perspective-taking and empathic-concern between the pre- and postassessment, as well as an inverse correlation between the empathy subscale of personal distress and assertiveness. It is concluded that students show adequate results in empathy and assertiveness. However, there is some influence of clinical practice on the development of empathy, and future intervention studies need to be considered. Furthermore, students with higher levels of assertiveness have lower levels of personal distress, suggesting that assertiveness is closely related to empathy.

## Introduction

Professional excellence in healthcare goes beyond technical competence; it requires the effective integration of skills that enable one to identify and understand situations, feelings, motives and perspectives and to recognise and value the concerns of others. These skills, such as empathy and assertiveness, are not only fundamental to the delivery of compassionate, patient-centred care but are also essential pillars in the education of health science students. Developing these skills not only strengthens the professional-patient relationship but also improves the quality of care by promoting a holistic approach that goes beyond the purely clinical to address the emotional and social needs of those cared for^1^.

A recent descriptive cross-sectional study^1^ showed that physiotherapy students at university have acceptable levels of empathy and assertiveness. Gender is closely related to empathic development, with females having the highest levels of empathic development. It was also noted that students from other health professions or with previous work experience had lower levels of empathic development in relation to personal distress.

Pastén Hidalgo et al.^2^, in a study also conducted with physiotherapy students, report that empathic development decreases or deteriorates in the last year of physiotherapy training. Regarding first-semester nursing students, Jérez Jaimes et al.^3^ show no significant differences in this student profile regarding gender, age, class or social origin. According to these authors, it should be considered that first-semester nursing students are still growing in terms of personal and empathic maturity. Martín Fernández^4^ points out that the level of empathy decreases with the development of a nurse or a medical professional and that, for this reason, empathy training should be included in the training of health professionals: a more empathetic professional will be less exhausted and will be able to provide better assistance to the patient. Burnout severity in medical professionals is associated with reduced empathy-related brain activity^5^. Among physiotherapists, higher levels of burnout have been shown to lead to lower levels of empathy. Burnout leads to a greater likelihood of personal discomfort and depersonalisation^6^.

On the other hand, Chu et al.^7^ found that female physiotherapy students showed better results in empathic development than their male colleagues. These authors highlight that male and female physiotherapy students further improve their levels of empathic development after participating in simulation activities, role-playing or dramatising situations that any patient might present. Grau et al.^8^ also found this among medical students in Spain: the empathic development of a group of medical students improved after having received training in communication and after having carried out simulations with role-playing activities. Sancho-Cantus et al.^9^ found it necessary for health science students to develop and improve their soft skills competencie; they show the benefits at the clinical level from students to patients and relatives in regard to having competencies in conflict resolution, teamwork and effective communication.

However, this requires health science students to grow internally during their education and to consider their perceptions of empathy and how this can be developed through mentors who model empathic behaviour, as Jobling and Alberti^10^ point out.

### Methods to improve health science students' empathy and other soft skills

Empathy between health care users and professionals contributes significantly to both groups' behaviour, therapy and general well-being. The development of empathic skills is an essential priority in the training of health and social care students. Educational programmes should be conducted in a practical way that strengthens students' personal and social skills and ability to communicate effectively with their patients^11^.

Research on service-learning (SL) in education has increased since the 1990s. In higher education physiotherapy programmes, this technique is used to achieve real hands-on learning and understanding of how to recognise and manage emotions, care for others and make decisions in clinical settings^12^.

Practical and reflective training to improve students' social and communication skills should be considered, as should the establishment of reliable and validated instruments to monitor the professional competence of future physiotherapists, as mentioned by Cobo Mejía et al.^13^. Angulo Casal^14^ says that it is also necessary to have a comprehensive assessment questionnaire of nontechnical skills. Kataoka et al.^15^ point out that improving communication between medical students through training improves empathy skills, although the authors say that further studies are needed. In general, simulation in physiotherapy has good results in training students at clinical and communication levels, as this also trains assertiveness^16^.

At present, the educational tool of virtual reality is achieving good results in the different curricula of different health disciplines, such as physiotherapy, nursing, medicine or psychology. Virtual reality helps students improve their understanding of patients with cognitive or visual impairment^17^. As noted by Morrow et al.^18^, artificial intelligence is becoming an innovative and effective tool to improve the care provided by healthcare professionals: it helps to improve compassion, empathic awareness, relational behaviour and clinical reasoning.

Regarding artificial intelligence, Gillespie et al.^19^ report that virtual reality simulation is achieving excellent results for learning and improving empathic development and attitudes and behaviours towards patients. Specifically in physiotherapy, Ward et al.^20^ report that virtual simulation helps improve empathic development at a cultural and interpersonal level in physiotherapy students. Taylor et al.^21^ also agree, arguing that video simulation helps to develop communication skills in physiotherapy students.

In general, simulation allows students in any health science discipline to learn meaningfully and not just by rote learning and even to communicate with the patient to convey and deliver bad news^22–24^. Furthermore, it has become a teaching strategy for any student training in any health science discipline or profession to ensure that the students are well-equipped with the necessary skills before caring for a patient^25^. However, clinical simulation requires other techniques, such as debriefing, reflection and the development of critical thinking for it to be transcendent at a pedagogical level among students^26^.

Medina Castellano et al.^27^ argue that using simulation with nursing students helps them identify ethical and legal problems in their professional practice and provide safe care. Caballero de la Calle^28^ has also shown that virtual reality can be a tool for training communication and preventing assaults on nursing students. The researcher Sánchez-Expósito^29^ agrees with the above author that nursing students who perform well in simulation show better performance and use of their clinical practice, obtaining better socioemotional skills. Mori et al.^30^, in a review, say that simulation is becoming a common pedagogical practice in training future physiotherapy professionals to develop all competencies. Simulation helps to develop nurses' empathy, security and self-confidence, as suggested by Ehibhatiomhan et al.^31^.

Other tools for developing and improving life and social skills include role-playing, the escape room, student clinical interviews with actors playing patients followed by a briefing, reflective practice or video feedback. Role-playing is an effective method to help students become aware of their interpersonal communication skills to develop emotional intelligence^7,32–35^. Regarding video viewing, Ahmadzadeh et al.^36^ explain that watching health-themed films improves medical students' empathy development and communication skills.

In an integrative review, Silva et al.^37^ state that most studies conducted among nursing and medical students use active teaching methods, such as literature, videos, the so-called workshop, case simulations or learning in real professional scenarios. Lozoya Angulo et al.^38^ also confirm the pedagogical effectiveness of active methodologies, as they promote comprehensive training.

Ultimately, however, as Menezes et al.^39^ point out that in order to mitigate or prevent the erosion of students' empathic and/or compassionate levels, sustainable teaching and training are needed throughout the entire undergraduate education and not just in a single training activity.

### Influence of clinical placements on soft skills development in health science students

Regarding clinical experience, Taveira-Gomez et al.^40^ report that medical students improved their competencies and communication skills after clinical placements. Yu et al.^41^ show an improvement in empathy levels among students who have experienced clinical placements compared to those who have not. However, these authors note that as this is a cross-sectional study, longitudinal studies are needed to confirm these findings further.

Imperato and Strano Paul^42^ show that activities can also be planned or trained during clinical placements to improve or train empathy. By setting up meetings and reflection rounds where medical students shared their feelings, experiences and reflections on their clinical placements, these students improved their empathic thinking compared to before they started their placements. On the other hand, Strohbehn et al.^43^ suggest visiting art exhibitions as an activity to develop empathic thinking in medical students during their clinical placements: they show an improvement in empathic thinking, although further research is needed in this respect. Ardenghi et al.^44^ state that, in general, extracurricular activities promoting self-awareness, self-reflection and communication can act as pedagogical models that enhance empathic thinking among students.

As there are few studies, at least with physiotherapy students, the influence of clinical practice on the development of empathy is an area to be studied.

Percy and Richardson^45^ say that teaching empathetic and compassionate care in nursing is a central theme on which the education of future professionals should be built. Güven et al.^46^ agree that assertiveness and empathy are necessary to educate a good nurse, as the high development of these two social skills develops better altruistic thinking. Improving empathic communication improves future nurses' emotional intelligence, leading to a better quality of care^4,47^. Kim and Shin^48^ show that the more competent a nursing student is at the socioemotional level, the better their academic and clinical performance. Regarding medical students, Chew et al. ^49^ also observed a correlation between emotional intelligence and academic performance.

### Justification of the importance of empathy and other soft skills in the training of physiotherapists and their relation to bioethics. Purpose of this study

Communication skills should be included in the formal undergraduate physiotherapy curriculum as they help improve the care provided by future physiotherapy graduates^50^. However, as mentioned by Utbaer et al.^51^, the role of teachers in supporting and empathising with students is essential for students to improve their professional competencies.

Assertiveness is also an essential social skill for both nursing and physiotherapy because, like empathy, it enables professionals to provide better quality care because they are better trained to overcome difficulties and conflicts in interpersonal relationships^52^. Luna et al.^53^ showed that nursing students with low levels of assertiveness may have a more significant development of anxiety and/or depression. It is, therefore, clear that the higher the level of assertiveness, the higher the level of self-esteem^54^. This correlates with the study by Hernández-Xumet et al.^1^, in which it was observed that, among physiotherapy students, those with low scores in assertiveness development were those with higher scores at the empathic level in the area of personal distress (PD), which includes the negative feelings, anxiety, unease and personal discomfort generated when empathising with others.

It is important to emphasise, as Nortjé et al.^55^ do that a healthcare professional should expect to develop some important virtues, such as compassion (actively understanding another person's perspective on their well-being with a deep understanding of their pain or difficulties) and the virtue of discernment (discernment involves the ability to make decisions free from the undue influence of personal interests or superficial concerns). Another is trust (an assertive virtue that implies the certainty that another will act with the right intentions and follow appropriate moral standards). Trust is an essential element, for example, in choosing a health professional. All these virtues are important components in the ethical development of a professional. As Gerace argues^56^, sympathy, empathy and compassion are terms that are often difficult for health professionals to define and explain in their helping relationships with patients.

The Spanish professor of ethics, Adela Cortina^57^, considers that a good professional must have a great sensitivity towards others; otherwise, he or she cannot be considered a good professional. Along with Cortina's ideas, Nishanthi and Vimal^58^ consider that a professional should be characterised by altruism and follow a moral code, which is why these authors advocate that bioethics should be studied in curricula since bioethics and professionalism are inextricably linked. Hernández-Xumet et al. show that the training of health professionals should integrate general knowledge common to all health professions, such as anatomy, physiology, pathology and research, as well as knowledge specific to each profession, medicine, nursing, physiotherapy and others. In addition, this training must also integrate interpersonal skills, ethics and bioethics to enhance the overall training of future health professionals. Therefore, the training of students is a process that synergistically integrates academic and clinical knowledge with a knowledge acquisition plan in academic and health centres, with professionals trained for the integrated implementation of all these competencies (relationship between theory and clinical practice, problem-solving, teamwork, critical thinking, responsibility and cultural competence)^1^.

Marqués-Sule et al.^59^ argue that the teaching of ethics should be established in each year of the university degree, even if it is transversal in each subject. Ethics is a fundamental pillar in the clinical training of physiotherapy students, and as Moreno-Segura et al.^60^ say, the human sensitivity of physiotherapy students should be explored to shape the design of ethics training in the physiotherapy curriculum, as ethics training undoubtedly also serves to train future professionals in patient safety^61^. Aguilar-Rodríguez et al.^59^ reinforce the idea of the importance of ethics training for future physiotherapists, as it will enable them to identify situations in clinical practice in which bioethical principles may be violated in patient care during their clinical training. As Marques-Sule et al.^61^ concluded, in general, future physiotherapists attach great importance to ethics training and its impact on their professional training. Ethical awareness is related to safety in clinical practice.

Considering the precedents of works that have studied soft skills in physiotherapy students and students of other disciplines, such as nursing and medicine, the authors understand the importance of studying their empathic and assertive development after experiencing the reality of care through the clinical practices of the fourth academic year to improve the training of future physiotherapy professionals. Soft skills are virtues and essential components in developing a physiotherapy professional to form critical and ethical thinking. Costa et al.^62^ emphasise the importance of bioethics in physiotherapy, especially in relation to end-of-life care.

**Figure 1:**
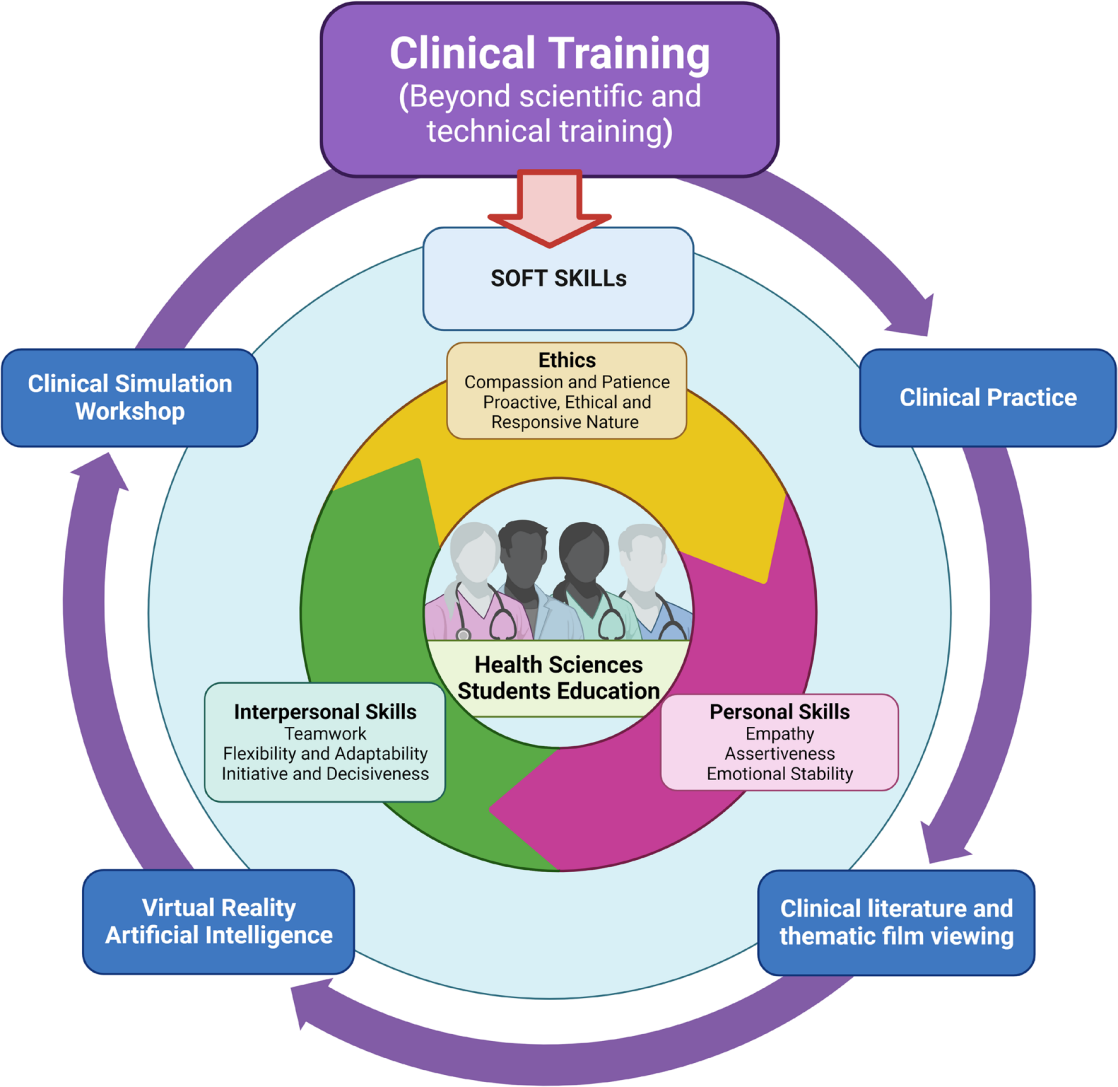
The scheme shows how Soft Skills are essential for health science professionals/students as they go beyond patient care and include characteristics such as character, behaviour, social skills and attitudes. Soft skills complement hard skills and can improve clinical outcomes for patients. A good healthcare professional communicates in a way that puts patients at ease, which can help to reduce anxiety and worry. Maintaining a positive outlook and a supportive atmosphere can even help patients with serious illnesses to recover. Healthcare staff empathy and assertiveness are critical to improving patient clinical outcomes. The clinical training of a health science student should be based on more than clinical practice. However, it should be included in a training programme that includes other aspects of practice that directly impact the better development of soft skills. Created with BioRender.com.

Therefore, this work aims to determine the empathic and assertive levels of fourth-year physiotherapy students at the university after clinical practice and to compare the results before and after the natural development of the course at the university. Based on the work of Yu et al.^41^, they suggest that students' empathy levels improve during clinical practice. The knowledge of the reality of our students allows us to plan future actions, a deeper exploration of our reality and specific adaptation actions. This work was conceived with the idea in mind that clinical training for health science students should go beyond scientific and technical training. See Fig. 1.

## Materials and method

A longitudinal and descriptive study study design was proposed following the STROBE guidelines. This study was approved by the University Research Ethics Committee (CEIBA-Universidad de La Laguna), with code CEIBA2022-3133.

The study was conducted between November 2022 and June 2023.

All students were recruited voluntarily and were free to withdraw from the study at any time. No participants were coerced or pressured to complete the surveys. They provided their informed consent for participation in the study in both pre-and postevaluations. The Declaration of Helsinki was followed throughout the research process.

### Research setting and participants

#### Context

The physiotherapy degree at the Universidad de La Laguna (ULL) consists of four academic years of 60 ECTS each. The fourth year of the physiotherapy degree consists of 40 ECTS of clinical placements, of which 750 h are preprofessional placements. The rest of the academic year corresponds to the final degree project (8 ECTS) and elective subjects (12 ECTS).

The physiotherapy degree at this university is a professional degree with a scientific but human vocation.

#### Participants

The study population was the fourth-year students of the physiotherapy degree programme at the university in 2022/2023. The total population of students in this year of the course was n = 65.

The inclusion criteria were as follows: (a) university physiotherapy students, (b) fourth-year students enrolled in clinical practice subjects (core subjects passed), and (c) students who consented to participate in the study with full knowledge of its purpose and content. Students had to meet the criteria of 1 and 2 above to be included in the study.

The exclusion criteria were as follows: (a) external university students on a national or international exchange programme that is, Erasmus, Sicue or similar; the Erasmus Programme is a student exchange programme between European universities; and the Sicue Programme is an exchange programme similar to Erasmus but between universities in Spain. In these programmes, students stay 6–12 months at the university).

#### Data collection

The study population was fourth-year students of the physiotherapy degree program at the university in 2022/2023. The total population of students in this year of the course was n = 65.

This longitudinal observational study consisted of five phases in which two student assessments were carried out: an assessment of empathy and assertiveness at the beginning of the course (pre) and another at the end of the course, after their clinical placement (post).

Stage 1 (information one pre) was an information stage where the teachers and researchers responsible for the study met with student representatives. The study's aims and all the information about the study were explained at this meeting: inclusion and exclusion criteria, informed consent, data protection and ethical considerations. All this information was sent to all fourth-year students via their institutional university email addresses. The questionnaires to be completed were also sent out in November 2022.

Thus, with the sending of the forms to be completed via the Google Forms platform, stage 2 (evaluation 1—pre) began in November 2022, when the preliminary evaluation of these students' empathic and assertive development was performed before they started their clinical practice. All forms were completed correctly and returned without incident. A total of 60 people (92.31%) completed the questionnaires.

Stage 3 (clinical practice, 750 h) corresponds to the student's clinical practice period. Students complete four rotations in public and private hospitals, primary health care centres, public administration social health centres, foundations and associations. They complete 750 h of clinical practice, equivalent to 40 ECTS, in the fourth year of study. This means that the internship represents 66.66% of the ECTS credits of this year, according to the curriculum of the physiotherapy degree at the university. The students do the clinical practice between October and May.

The second evaluation of the students' empathic and assertive levels was performed after the clinical placement. This corresponds to stage 4 (evaluation 2—post). The students completed the questionnaires again, and all the information was sent to their institutional university e-mail addresses. As in the preevaluation, all questionnaires were duly completed by the participants. Only 51 of the original 60 people participated in this postevaluation (attrition rate = 15%).

**Figure 2:**
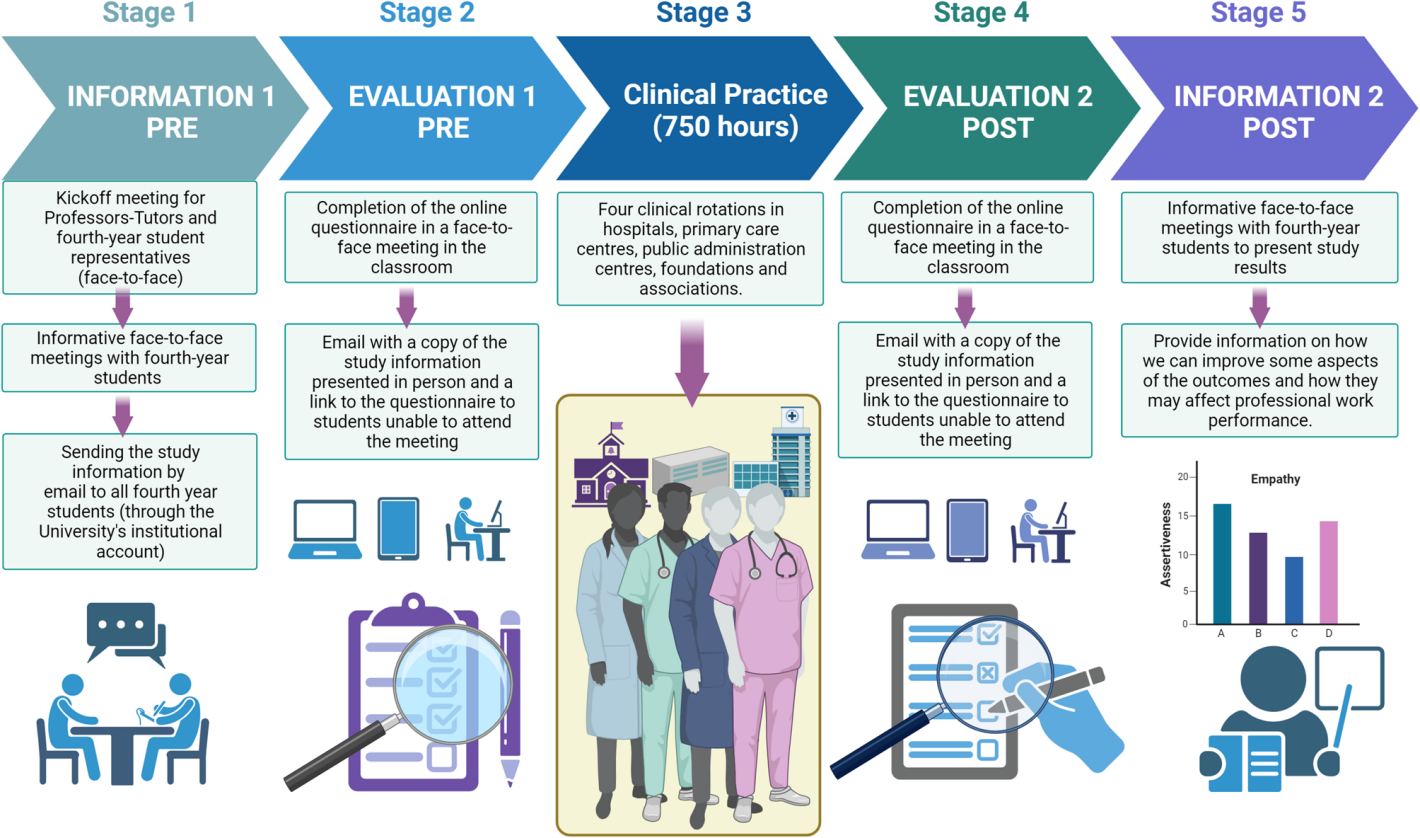
The scheme shows the process of student selection and how data are acquired. The working protocol is developed in five phases: the first phase consists of providing information about the study; the second phase consists of data collection; in the third phase, students assist in clinical practice; the fourth phase consists of data collection (final evaluation); the fifth phase consists of providing information to students on how we can improve some aspects of the results and how they may affect professional work performance. Created with BioRender.com.

After analysing the data from the pre-and postevaluation of clinical practice, a meeting was held with the participating students to present and explain the results and how they can be interpreted for their professional practice. This information meeting in June corresponds to stage 5 (information two post). See Fig. 2.

#### Measurement instruments

In order to assess the empathic and assertive development of these students, the following instruments were used and are described below.

##### Interpersonal reactivity index (IRI-Spanish version)^63,64^

The IRI empathy questionnaire is an essential tool in research because it provides a standardised way to measure different dimensions of empathy. Empathy is a complex construct that involves understanding and responding to the emotions and experiences of others. It has links to many important outcomes, such as social competence, relationship quality, and mental health.

The IRI is a measure of dispositional empathy that takes as its starting point the notion that empathy consists of a set of separate but related constructs^65,66^. The instrument contains four subscales, each covering a separate facet of empathy.

The four IRI subscales measure four dimensions of the global concept of empathy. Some show more cognitive aspects (the perspective taking-PT and fantasy-FS subscales). They are related to the spontaneous attempts of the subject to adopt the perspective of the other and to understand the point of view of the other person. They evaluate the tendency to identify with others and the imaginative capacity to put themselves in fictitious situations^63,65,66^.

The other two subscales, empathic concern (EC) and personal distress or discomfort (PD) measure people's emotional reactions to negative experiences. In the first (EC), the feelings “oriented towards the other person” are measured; in the second (PD), the feelings of anxiety and discomfort that the subject manifests when observing the negative experiences of others are evaluated (these are feelings “self-oriented”). Therefore, the two subscales refer to different feelings.

The IRI-Spanish version is one of the most widely used self-report measures for assessing students' empathy^63,64^. The IRI is, together with the Jefferson Scale of Physician (JSPE), one of the most common measurement instruments for the study of empathy^67^, and it has also been validated for the measurement of empathy among Spanish-speaking university students^68^. The reliability of the IRI Empathy Questionnaire ranges between 0.66 and 0.84 (Cronbach's *α* coefficients of the four subscales that make up the instrument)^64^.

##### The Rathus assertiveness scale (RAS-Spanish version)^69–72^

The RAS questionnaire provides a standardised way to measure an individual's level of assertiveness. Assertiveness is a communication style that involves expressing one's thoughts, feelings, and needs directly and respectfully. It is a critical social skill that can influence an individual's personal and professional relationships and overall well-being.

The RAS was designed to measure a person's level of assertiveness. It is also an instrument for measuring behavioural change in assertion training. The RAS was developed in 1973 by Spencer Rathus^72^. The RAS consists of 30 items (including 16 inverted items) with a 7-point Likert scale scored from − 3 (very uncharacteristic of me) to 3 (very characteristic of me). Total scores range from − 90 to 90 points and provide a score for interpretation. The RAS result can also be divided into three intervals: (a) Very assertive (from 30 to 90), (b) Acceptable assertiveness (from − 30 to 30), and (c) Slightly assertive (from − 90 to − 30).

The reliability of the RAS Questionnaire ranges between 0.73 and 0.86 (Cronbach's *α* coefficient)^71^.

### Data analysis

Data management and analysis were performed using SPSS 26.0 (IBM, 2019) and Jamovi version 2.3.17 (Project, 2023). Descriptive analyses were conducted, and mixed ANOVAs were conducted with the intragroup factor (pre/post) and the intergroup factor gender (male/female) to test whether gender or clinical practice affected empathy or assertiveness. The relationship between empathy and assertiveness scales was analysed using Pearson correlation analysis. A *p*-value ≤ 0.05 was considered statistically significant.

### Institutional review board statement

The study was approved by the Universidad de La Laguna Research Ethics Committee (CEIBA‐ ULL), with code CEIBA 2022‐3133. The study was conducted according to the guidelines of the Declaration of Helsinki. Informed consent was obtained from all subjects involved in the study.

### Informed consent

Informed consent was obtained from all subjects involved in the study. Informed consent was obtained from the students to publish this paper.

## Results

A total of 60 people (92.31% of the population meeting the inclusion and non-exclusion criteria) received the information and completed the first questionnaire. Only 51 of the original 60 people participated in the second questionnaire (attrition rate = 15%; five students from our university participated in an exchange programme with another university, and four people did not respond to the second questionnaire). For the analysis of the results and the pre- and post-comparison, only the data of the 51 participants in the pre and post-measurement were taken into account.

The sociodemographic data of the sample are described first. Then, the descriptive data from the first and second assessments are explained, followed by the interferential analysis. Finally, the correlational data from the pre-and postassessments are presented.

### Descriptive analysis of the sample

Fifty-one out of 65 students (78.5%) participated in the total study, and 51 questionnaires were received in the final analysis. All questionnaires completed by the students were valid; there were no partially completed questionnaires or missing data.

All participants were aged between 21 and 42 years (M = 22.59; SD = 3.57). The sample consisted of 28 females and 23 males.

#### Descriptive data first evaluation-pre: IRI and RAS

The empathy subscale perspective taking (PT) had a higher score than the other subscales (M = 27.86; SD = 3.82), followed by empathic concern (EC) (M = 26.98; SD = 3.26). The fantasy (FS) subscale (M = 17.94; SD = 3.62) was third and the scale with the lowest score was personal distress (PD) (M = 17.04; SD = 3.89). The assertiveness score (RAS) was acceptable (M = − 7.18; SD = 24.25). See Table 1 below for more details by gender.

**Table 1:** Interpersonal Reactivity Index (IRI) and Rathus (RAS) score in students of physiotherapy. First evaluation-pre.

		PT_perspective taking	FS_fantasy	EC_empathic concern	PD_ personal distress	RAS‐students
		Median/SD	Median/SD	Median/SD	Median/SD	Median/SD
Score (TOTAL)		27.86/3.82	17.94/3.62	26.98/3.26	17.04/3.89	− 7.18/24.25
Score (Gender)	Female	28.46/2.96	18.43/3.82	27.7/2.65	16.79/4.06	− 9.18/24.40
	Male	27.13/4.62	17.35/3.35	26.09/3.75	17.35/3.75	− 4.74/24.38
Cronbach's α		0.78	0.67	0.55	0.63	0.87

#### Descriptive data second evaluation-post: IRI and RAS.

In the second assessment of the IRI subscales and the RAS scale, the highest total score was in the EC subscale (M = 26.80; SD = 4.66), followed by the PT subscale (M = 26.33; SD = 5.14), FS (M = 18.61; SD = 3.68) and PD (M = 16.86; SD = 3.93). The assertiveness scale (RAS) was also acceptable in the second assessment (M = − 6.94; SD = 20.39). See Table 2 below for further details by gender.

**Table 2:** Interpersonal Reactivity Index (IRI) score in students of physiotherapy. Second evaluation-post.

		PT_perspective taking	FS_fantasy	EC_empathic concern	PD_ personal distress	RAS‐students
		Median/SD	Median/SD	Median/SD	Median/SD	Median/SD
Score (TOTAL)		26.33/5.14	18.61/3.68	26.80/4.66	16.86/3.93	− 6.94/20.39
Score (gender)	Female	27.82/4.87	19.64/4.34	29.21/3.51	16.43/4.18	− 9.11/20.06
	Male	24.52/4.97	17.35/2.17	23.87/4.27	17.39/3.63	− 4.30/20.92
Cronbach's α		0.70	0.79	0.63	0.69	0.86

### Inferential analysis

A mixed ANOVA analysis was performed with an intrafactor (pre/post) and an interfactor (gender–males/females). The main effect pre-post was statistically significant in the PT subscale (F_1,49_ = 9.32; *p* = 0.004; η^2^ = 0.03); and the main effect of gender in PT (F_1,49_ = 4.32; *p* = 0.043; η2 = 0.06); see Sections "Empathy subscales Fantasy and Personal Distress, and Assertiveness." and "Empathy Subscale Perspective Taking"; and Table 3 below. In addition, the interaction of pre/post by gender was significant in EC (F_1,49_ = 18.55; *p* < 0.001; η^2^ = 0.05); see Section "Empathy subscales Fantasy and Personal Distress, and Assertiveness." and Table 3.

**Table 3:** Inferential analysis—Empathy Subscales/Assertiveness by pre-post.

	pre-post	n	Mean	SD	F	Sig	η^2^
PT_perspective taking	Pre	51	27.86	3.82	F_1,49_ = 9.32	*p* = 0.004*	0.03
	Post	51	26.33	5.14			
FS_fantasy	Pre	51	17.94	3.62	F_1,49_ = 1.75	*p* = 0.192	0.01
	Post	51	18.61	3.68			
EC_empathic concern	Pre	51	26.98	3.26	F_1,49_ = 0.69	*p* = 0.410	0.00
	Post	51	26.80	4.68			
PD_ personal distress	Pre	51	17.04	3.89	F_1,49_ = 0.11	*p* = 0.739	0.00
	Post	51	16.86	3.93			
RAS‐students	Pre	51	− 7.18	24.25	F_1,49_ = 0.02	*p* = 0.880	0.00
	Post	51	− 6.94	20.39			

*p ≤ .05.

#### Data from the inferential analysis—empathy subscales/assertiveness by pre-post

**Figure 3:**
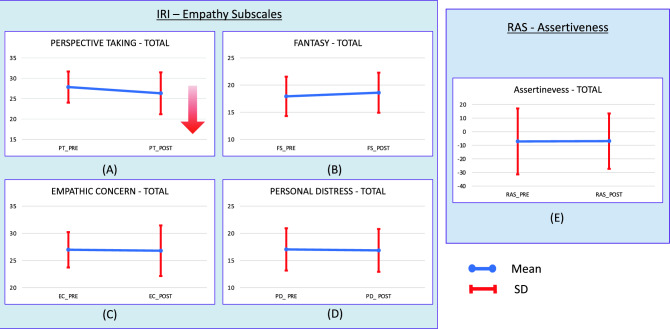
The figure shows the behaviour of the empathy subscales and assertiveness compared between pre-post moments. There is a statistically significant difference in the perspective taking subscale (**A**). There is a higher score in PT at the pre time than at the post time. The other empathy subscales (in the figure as (**B**), (**C**) and (**D**)) and the assertiveness (in the figure as (**E**)) show no differences between pre and post.

In the inferential study of the empathy subscales and assertiveness comparing pre-post moments, a statistically significant difference was observed in the perspective taking (PT) subscale (F_1,49_ = 9.32; *p* = 0.004; η^2^ = 0.03). There was a higher score in PT in the pre time (M = 27.86; 3.82) than in the post time (M = 26.33; SD = 5.14). The remaining empathy subscales and assertiveness (RAS) showed no differences between pre and post. See Fig. 3 and Table 3 for more details below.

#### Inferential Analysis. Empathy subscales/Assertiveness by gender

There were significant gender differences in PT (F_1,49_ = 3.41; *p* = 0.043; η2 = 0.06). Females scored higher than males overall (female M = 28.14; SD = 3.92///male M = 25.83; SD = 4.80).

There were significant gender differences in EC (F_1,49_ = 15.00; *p* < 0.001; η^2^ = 0.16). Females scored higher overall than males (female M = 28.46; SD = 3.08///male M = 24.98; SD = 4.01). This higher posterior score in females can also be seen in the final score (t_1,49_ = 4.91; *p* < 0.001; r2 = 0.33 /// Female M = 29.21; SD = 3.51///Male M = 23.87; SD = 4.27).

#### Inferential analysis. Empathy subscales/assertiveness by pre-post/gender

**Figure 4:**
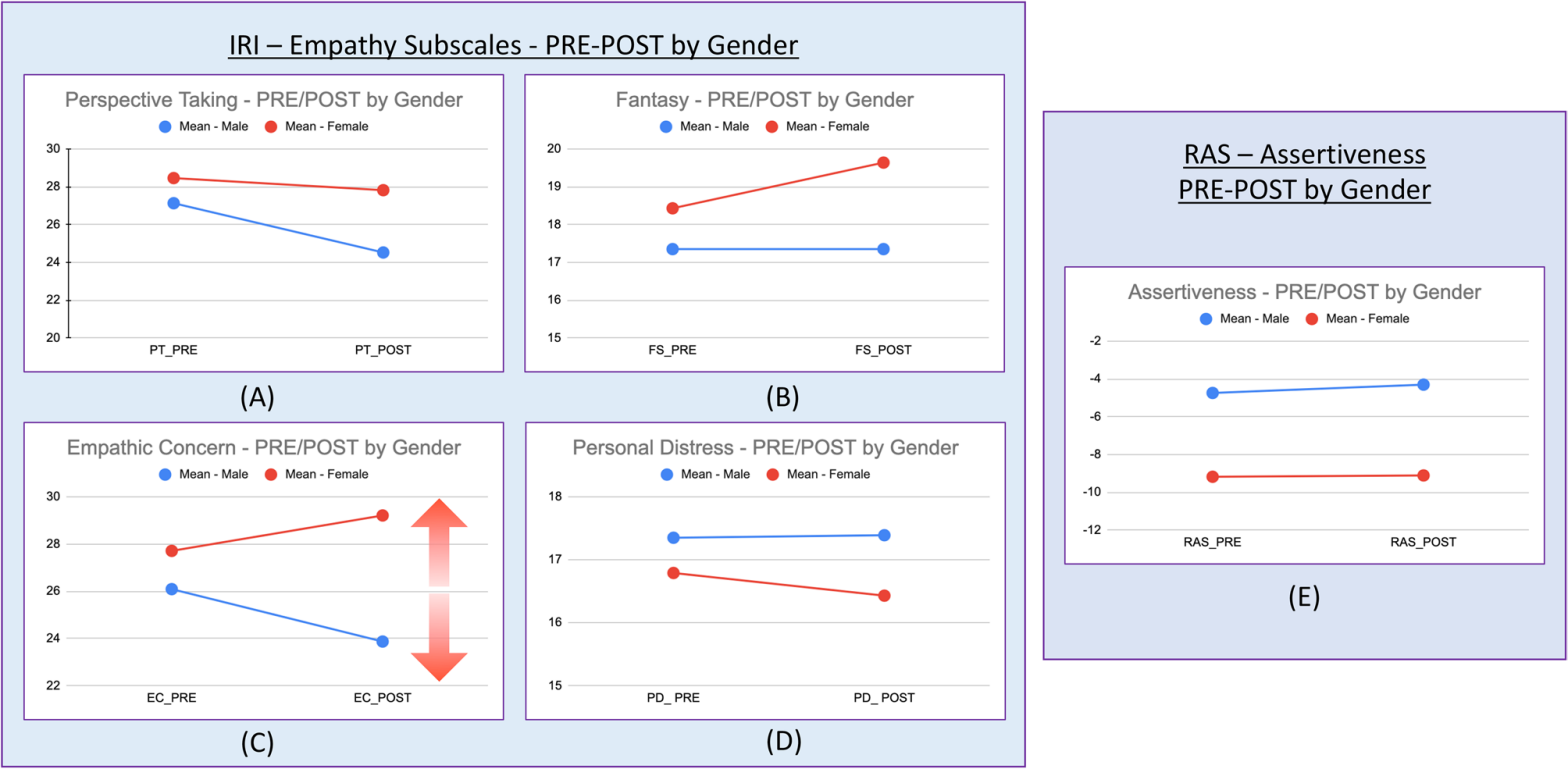
The figure shows the behaviour of the empathy subscales and assertiveness between the pre-post moments by gender. We observe a significant difference between women and men in the empathic concern variable at the post time (**C**): women have higher scores than men. A significant decrease in the empathic concern score is observed for men (**C**) in the final evaluation. There are no significant differences in the other empathy subscales (in the figure as (**A**), (**B**) and (**D**)) and in assertiveness (in the figure as (**E**)).

Regarding the inferential analysis between empathy subscales and assertiveness (RAS), higher scores were observed for women on the PT and EC subscales, with a significant difference between women and men for the empathic concern variable in post: the women´s score was M = 29.21; SD = 3.51 and the men´s score was M = 26.09; SD = 3.75. Thus, there is a significant decrease in men's empathic concern (EC) scores. In the subscales fantasy (FS) and personal distress (PD), there were no significant differences in the interaction. See Fig. 4 and Table 4 below for more details.

**Table 4:** Inferential analysis—Empathy Subscales/Assertiveness by pre-post/Gender.

	Gender	Pre-post	n	Mean	SD	F	Sig	Effect
PT_perspective taking	Female	Pre	28	28.46	2.96	F_1,49_ = 3.41	*p* = 0.071	**η**^**2**^ = 0.01
		Post	28	27.82	4.87			
	Male	Pre	23	27.13	4.62			
		Post	23	24.52	4.97			
FS_fantasy	Female	Pre	28	18.43	3.82	F_1,49_ = 1.75	*p* = 0.192	**η**^**2**^ = 0.01
		Post	28	19.64	4.34			
	Male	Pre	23	17.35	3.35			
		Post	23	17.35	2.17			
EC_empathic concern	Female	Pre	28	27.71	2.65	F_1,49_ = 18.55	*P* < 0.001*	**η**^**2**^ = 0.05
		Post	28	29.21	3.51			
	Male	Pre	23	26.09	3.75			
		Post	23	23.87	4.27			
PD_personal distress	Female	Pre	28	16.79	4.06	F_1,49_ = .18	*p* =` 0.739	**η**^**2**^ = 0.00
		Post	28	16.43	4.18			
	Male	Pre	23	17.35	3.75			
		Post	23	17.39	3.63			
RAS‐students	Female	Pre	28	− 9.18	24.40	F_1,49_ = .01	*p* = 0.913	**η**^**2**^ = 0.00
		Post	28	− 9.11	20.06			
	Male	Pre	23	− 4.74	24.38			
		Post	23	− 4.30	20.92			

*p ≤ .05.

### Correlational analysis

#### Correlation analysis—empathy subscale and assertiveness. First assessment-pre

This correlational study of the empathy subscales and the assertiveness of the first assessment-pre is worth highlighting because of the significant correlation between the empathy subscales perspective taking (PT) and empathic concern (EC), sharing 29.16% of their variance (r = 0.54; *p* < 0.001) and the statistical significance between the Assertiveness scale (RAS variable) and the personal distress (PD) subscale, sharing 25% of their variance (r = − 0.50; *p* < 0.001). See Table 5 below for more details.

**Table 5:** Correlation analysis—Empathy Subscale and Assertiveness. First evaluation-pre.

		PT_perspective taking	FS_fantasy	EC_empathic concern	PD_ personal distress	RAS‐students
PT_perspective taking	Pearson´s R	–	–	–	–	–
	*p* value					
FS_fantasy	Pearson´s R	0.17	–	–	–	–
	*p* value	*p* = 0.233				
EC_empathic concern	Pearson´s R	0.54	0.26	–	–	–
	*p* value	*p* < 0.001*	*p* = 0.071			
PD_ personal distress	Pearson´s R	− 0.04	0.11	0.01	–	–
	*p* value	*p* = 0.788	*p* = 0.457	*p* = 0.947		
RAS‐students	Pearson´s R	− 0.16	− 0.01	− 0.09	− 0.50	–
	*p* value	*p* = 0.253	*p* = 0.944	*p* = 0.527	*p* < 0.001*	

*p ≤ .05.

#### Correlational analysis—empathy subscales and assertiveness. Second evaluation-post

In the correlation analysis of the empathy subscales and assertiveness of the second postassessment, statistical significance (*p* < 0.001) is observed between the empathy variables perspective taking (PT) and empathic concern (EC). Similarly, the relationship between the empathy variable personal distress (PD) and Assertiveness (RAS) also reached statistical significance (*p* = 0.001). The relationship between the perspective taking (PT) subscale and the fantasy (FS) (*p* = 0.007) and personal distress (PD) subscales (*p* = 0.045) reached statistical significance. The relationship between the FS and EC subscales also reached statistical significance (*p* = 0.007). See Table 6 below for more details.

**Table 6:** Correlation analysis—Empathy Subscales and Assertiveness. Second evaluation-post.

		PT_perspective taking	FS_fantasy	EC_empathic concern	PD_personal distress	RAS‐students
PT_perspective taking	Pearson´s R	–	–	–	–	–
	*p* value					
FS_fantasy	Pearson´s R	0.37	–	–	–	–
	*p* value	*p* = 0.007*				
EC_empathic concern	Pearson´s R	0.55	0.37	–	–	–
	*p* value	*p* < 0.001*	*p* = 0.007*			
PD_ personal distress	Pearson´s R	− 0.28	0.17	0.09	–	–
	*p* value	*p* = 0.045*	*p* = 0.244	*p* = 0.536		
RAS‐students	Pearson´s R	− 0.14	− 0.22	− 0.23	− 0.44	-
	*p* value	*p* = 0.341	*p* = 0.116	*p* = 0.098	*p* = 0.001*	

*p ≤ .05.

## Discussion

The present study compares the empathic and assertive development of final-year physiotherapy students. This year in the course is characterised by clinical placements accounting for 77% of their academic performance and an experience similar to that of a physiotherapy professional. The results show that students' levels of empathy and assertiveness are acceptable and that clinical placements have a significant impact on students' empathic skills. There is a decrease in some empathy subscales in the final assessment (PT and EC subscales) and differences in scores according to gender (PT and EC subscales). The rest of the empathy subscales (FS and PD) and the assertiveness score remained unchanged from the initial assessment.

The implications of these results are significant for the training of future physiotherapists. Firstly, students' levels of empathy and assertiveness are acceptable, these are essential skills for physiotherapists as they enable them to understand and respond to their patients' needs. Secondly, clinical placements allow students to practice their empathy skills in a real-life setting and help them develop these skills effectively, suggesting that this is a valuable experience for the professional development of physiotherapists and needs to be well planned for optimal results.

The decline in some empathy subscales in the final assessment may be due to several factors, such as fatigue, stress or the need to focus more on technical skills or clinical practice assessments. The differences in development according to gender are also interesting, as they suggest that women and men may experience empathy differently during the development of clinical practice.

The fact that the other empathy subscales (FS and PD) and the assertiveness score remain unchanged from the initial assessment may indicate that these skills are developed more gradually and that the clinical practices may have had an uneven impact on the different empathy and assertiveness subscales or that the students had experienced a qualitative change in their empathy and assertiveness process, as indicated by the new correlations that appear in the final assessment and were not present in the initial assessment.

These qualitative changes in correlations suggest that clinical placements helped the physiotherapy students develop a deeper understanding of their patients' emotions and a more effective ability to communicate their own needs and wishes.

In the first assessment, the direct correlation between scores on the perspective taking (PT) and empathic concern (EC) variables and an inverse correlation between scores on the Rathus and personal distress (PD) variables suggest that people who are good at understanding and empathising with others are also more likely to be assertive and to experience less personal distress. These correlations have important implications for understanding and developing empathy, assertiveness and psychological well-being.

In the final assessment, positive correlations were found between perspective taking (PT) and empathic concern (EC) and between fantasy (FS) and empathic concern (EC), suggesting that empathy is a multidimensional construct related to the ability to put oneself in the place of others, the ability to feel empathy for others, and the ability to imagine oneself in situations involving others. The inverse correlations between perspective taking and personal distress and between Rathus and personal distress, suggest that empathy and self-esteem are essential factors in psychological well-being.

The data and correlations suggest that the clinical placements helped the physiotherapy students develop their cognitive and emotional skills of empathy and assertiveness. These qualitative changes could be essential to the success of physiotherapists, enabling them to build strong relationships with their patients and provide suitable care. Specifically, the clinical placements appear to have helped students to:

- Improve their ability to understand the emotions of others.
- Express themselves more assertively.
- Put themselves in other people's shoes and understand their needs.

This qualitative change in the students' empathic and assertive process may be due to a combination of factors, including the following:

- Exposure to a wide range of patients with different needs and experiences.
- The opportunity to practice their empathy and assertiveness skills in a real-life setting.
- Feedback from tutors and patients.

However, the personal discomfort students felt in response to others' emotions was still a factor that made it difficult for them to express themselves assertively. Therefore, it may be interesting to develop specific training programmes on personal distress management. Such programmes could help students develop strategies to manage the personal distress they experience in response to the emotions of others. Rodríguez-Nogueira et al.^73^ studied how personal distress influences empathy, patient care and the therapeutic alliance.

There is a paucity of literature on longitudinal studies of empathic and assertive development, especially the latter.

The results obtained are analyzed below, taking into account previously published work and following the chronological order of the present study.

### Descriptive data from the initial assessment and correlations

Over the last few decades, studies have been published which that have found that different types of empathy are associated with different cognitive and neurobiological processes. This research suggests that empathy is a complex construct involving different cognitive, neurobiological and personal developmental processes. The said research also suggests that different types of empathy may have different functions in social and emotional life^3,65,66,74,75^.

Regarding the empathy scores with the assertiveness scores, a significant correlation was observed between the personal distress subscale (PD) and the assertiveness scores (RAS). In both the pre- and postassessments an inverse correlation was obtained between these two variables, which would indicate that the higher the level of assertiveness, the lower the personal distress (PD) score. This means that there is a lower risk of empathic decline among students and better communication and a lower risk of anxiety/depression^52^, Luna et al.^53^ and Valdivia et al.^54^. Authors such as Güven et al.^46^ and Percy and Richardson^45^ argue, in the case of nursing students, that assertiveness and empathy are two essential foundations for preparing a nursing professional.

One possible explanation is that assertiveness is related to self-esteem and psychological well-being. In other words, people who feel confident and have a good self-image are less likely to experience personal discomfort. Another possibility is that assertiveness is related to the ability to cope with stress and anxiety. In this case, assertive people are more likely to have healthy coping strategies, which may help them manage stress and anxiety more effectively, as has been reported in other studies mentioned above^1,53,54^.

Carvalho et al.^76^ show that emotional intelligence is related to the well-being of medical, nursing and physiotherapy students. If students in these professions have good well-being, they will provide better care to their future patients and burnout syndrome can be prevented. Delgado et al.^77^ recently observed a relationship between burnout and specific areas of empathy in health professionals, linking depersonalisation with higher levels of personal distress and lower levels of empathic concern. This latter idea of Delgado et al.^77^ is consistent with the argument of Razi et al.^78^ who say that a decline in empathy has implications for burnout in health professionals and the safety of patient care. Regarding empathy and patient care, a strong relationship has been found between good empathy scores and therapeutic alliance among physiotherapists, as noted by Rodríguez-Nogueira et al.^73^. There is also a strong relationship between empathic development and burnout, as mentioned above, at both somatic and cognitive-behavioural levels^5,77^. Therefore, there is a correlation between empathy, self-actualisation, burnout and appropriate patient care.

The authors see a need for their students to be trained in soft skills such as assertiveness, as there is a strong correlation between empathy, assertiveness and the risk of psychological disorders and burnout and thus improving these skills would help them to provide better care for their future patients.

### Development of variables during the course

#### Empathy subscales fantasy and personal distress, and assertiveness

The comparison shows no significant differences in these variables between the initial and final assessments. This suggests that the clinical placements did not have a significant impact on the physiotherapy students' empathy and assertiveness skills for the following variables:

- Personal Distress (PD): the ability to feel anxiety and discomfort in the face of negative emotions in others^63–66^.
- Empathic Fantasy (EF): the ability to identify with fictional characters and their emotions^63–66^.
- Rathus' assertiveness: the ability to express one's opinion clearly and respectfully^69,70,72^.

There are several possible explanations for this finding. One is that the clinical placements were not long or intensive enough to significantly impact these skills. Another possibility is that the empathy and assertiveness skills of the physiotherapy students were already well developed at the beginning of the course; in this regard, they are acceptable, as noted in the previous section, and that the observational and cross-sectional research carried out by Hernández-Xumet et al.^1^ on physiotherapy students from all courses found that the students presented acceptable levels of empathy and assertiveness.

Further research is needed to better understand the impact of clinical practice on physiotherapy students' empathy and assertiveness. This research should use more sensitive assessment methods, e.g., longitudinal analyses, cohort studies, and even other qualitative or mixed research study tools. The authors agree with Yu et al.^41^ regarding longitudinal studies and educational intervention studies such as those conducted by Chu et al.^7^ or Grau et al.^8^.

#### Empathy subscale perspective taking

Significant differences were also observed between the initial and final assessments in the perspective-taking subscale (PT).

In this case, the clinical placements would not have positively affected the physiotherapy students' ability to put themselves in the shoes of others. In this context, other studies report that clinical placements contribute to developing empathy and other communication skills^40,41^. It is also true that these skills are improved during clinical placements when purposeful activities are planned^42–44^. Some studies find a decrease in empathy among health science students as they progress through their training^2–4^.

There are several possible explanations for this finding of lower scores. One possibility is that the clinical placements were too challenging or stressful for the physiotherapy students, which caused them to become emotionally distant from their patients and have difficulty understanding their emotions.

Another possibility could be that the clinical placements were not sufficiently structured and supported. This could have left physiotherapy students needing more guidance and feedback to develop their empathy skills. In any case, the lower score on the PT subscale suggests that clinical placements are not always a positive experience for developing empathy in physiotherapy students. Some possible suggestions for improving the outcomes of clinical placements on empathy development are as below:

- Provide physiotherapy students with the opportunity to interact with patients with a wide range of experiences and perspectives. This will help them develop a greater understanding of the emotions and feelings of others.
- Provide physiotherapy students with feedback on their empathy skills during the course. This will help them to identify their strengths and weaknesses and develop strategies to improve their skills.
- Structure clinical placements so that they are challenging but also supportive. This will help student physiotherapists develop empathy skills positively.

It should not be overlooked that in the inferential analyses here, women score much higher on perspective taking (PT) than men. Evolutionarily speaking, at the neurobiological level, women show a higher development of empathy at the phylogenetic and ontogenetic levels. One explanation may be that throughout evolutionary history, women have always been the protectors and caregivers of their children from when they were babies. Women are, therefore, more sensitive than men. Men, on the other hand, are neurobiologically more developed in the area of cognitive empathy^79^.

As educators, we need to help ensure that clinical practice is a valuable experience for developing empathy in every student physiotherapist.

#### Empathy subscale empathic concern

There are significant differences in the EC variable: between men (final score is lower than initial score); in the final measures between men and women (women's score is higher than men's score—in the initial assessment, there were no significant differences between men and women); and between men's and women's total scores (women's score is higher than men's score). There is a tendency for the women's final score to increase from the initial score (close to statistical significance).

In the interferential analysis between males and females, worse empathic concern scores were observed in males than in females, and it is necessary to investigate whether there is greater depersonalisation in male students as this may have implications for providing less humane care^78^. This suggests that clinical placements had a negative impact on men's ability to feel compassion, concern and caring in the face of others' discomfort, and the opposite for women. In addition, women have a more noteworthy natural ability to feel compassion and discomfort for others^79^. This is contradicted in the study by Moreno Segura et al.^60^. These authors argue that physiotherapists have an acceptable level of ethical and moral sensitivity. However, they note that women show better levels compared to men. According to the findings of the above researchers, understanding the characteristics of physiotherapists helps build ethical training for physiotherapists to improve clinical care. In the present study, the authors recognise this importance as empathy, if eroded, can affect sensitivity. We, as educators, need to be aware of possible deterioration in empathy levels, such as in the empathy EC subscale.

Clinical practice may have positively impacted women's ability to feel compassion and discomfort in others. Therefore, educators need to be open to studying and training empathy and other soft skills in their students and their perceptions of them. In addition to being role models for students in human attentiveness^9,10,39,51^. The authors mentioned above defend the idea that a health professional should be characterised by virtues such as compassion, sensitivity, empathy and altruism, as these are fundamental pillars for the development of a good health professional^1,55,57,80^.

### Descriptive final assessment data and correlations

In a longitudinal study with Turkish physiotherapy students, Yucel et al.^81^ show that empathy decreases in the fourth year. Empathy levels were found to be better among first-year students. The results in the present study found lower scores for perspective-taking (PT) and empathic concern (EC) after clinical practice in final-year students during the academic year. The authors also agree with the findings of Jerez Jaimes et al.^3^, who show that there is an empathic decline throughout the training of nurses and doctors, and with the results of Pastén Hidalgo et al.^2^, who also describe a decline in empathic development in physiotherapy students. Unlike the present study, which used the IRI, the studies by Yucel^81^, Jerez Jaimes et al.^3^, and Pastén Hidalgo et al.^2^ used the Jefferson test as an instrument to measure empathy.

However, in their cross-sectional study, Yu et al.^41^ suggest that medical students improved their empathic development during their clinical placements on the subscales of empathy (EC) (higher scores for females compared to males) and perspective-taking (PT). There were also good results on the Jefferson scale. These above authors suggested conducting longitudinal studies in clinical practice: in the case of the present study, with fourth-year physiotherapy students, significant differences were observed in perspective-taking (PT) and empathic concern (EC) among males. The authors agree with the above researchers on how the subsequent transition to clinical practice influences empathic development in EC. The clinical placement influenced the students in the present study, as described above.

Regarding gender, significant changes were also observed, as described above, with women scoring higher in empathy. Chu et al.^7^ also observed the same trend, with female physiotherapy students scoring higher on empathy than male students, even after an educational intervention. The same is argued by Grau et al.^8^ for medical students and by Deng et al.^82^, in this case nursing students, who agree that women show greater sensitivity and empathy than men. According to Deng et al.^82^, problem-solving skills have the most significant impact on the empathic development of nursing students. Health sciences educators need to recognise gender differences in empathy, emotional intelligence and problem-solving skills. Specifically, the results of the present study show promising results in the empathic and emotional levels as in the subscales of fantasy (FS) and perspective taking (PT) in the pre- and poststudy.

The results show that women score better than men on the three subscales of empathy, such as perspective taking (PT), empathic concern (EC) and fantasy (FS). However, the opposite is true for personal distress (PD) and RAS where men score higher than women. This can be interpreted in the sense that the greater the empathy is, the greater the emotional exhaustion. This was observed in the observational and cross-sectional study of physiotherapy students by Hernández-Xumet et al.^1^, where women scored higher on empathy than men and higher on personal distress (PD) than men.

The authors therefore understand that empathy and the development of other soft skills, such as assertiveness, are closely related to gender.

In other words, it is generally observed that students' empathy is influenced by clinical practice, and teachers need to assess its development and propose intervention activities to prevent the erosion of empathic development, as is argued below.

### Future research

Activities to develop empathic thinking and ensure that empathic thinking does not decline should be scheduled throughout the degree course. Simulation and video simulations are excellent tools to improve health science students' critical, empathic, ethical, and clinical thinking and thus improve and ensure better patient safety; they are effective pedagogical tools. Digital tools, information, and communication technologies for communication and empathy training should also be considered^7,27,28,30–35,38,39,42–44,83^.

According to Lee et al.^84^, empathy may be affected by current digital technologies because these may affect communication skills. The above authors advocate further research into the development of empathy in nursing students as it is affected in the digital age. Digital empathy is an emerging issue; nursing students should also be trained in digital empathy/communication skills. Alfonso-Mora et al.^16^ also agree that simulation is a tool to improve assertive communication in physiotherapy students.

Ward et al.^20^ point out that virtual simulation is another valuable and effective tool for the development of empathy in physiotherapy students, especially with cultural empathy demonstrated in a pre and post- (longitudinal) study. Therefore, there is a need to consider further longitudinal studies in the future, trying to implement interventions such as video simulation, as these researchers argue, and not just observational studies in a given period. Experimental pedagogical techniques have been shown to be more effective in empathy training than teaching and learning methods^85^.

We need to continue with studies and interventions to improve the soft skills of our students, such as the study conducted by Sung and Kweon^86^, which wasa pilot study among nursing students to assess empathy. Their results showed that the scores for self-esteem, empathy, interpersonal relations and communication skills were significantly higher in the experimental group than in the control group. Based on the findings of the said study, there is a need for continuous and ongoing education that can help nursing students improve empathy-based interpersonal relationships and communication skills within the nursing curriculum.

Grau et al.^8^, in a longitudinal study with an educational intervention, found that empathy on the perspective-taking (PT) subscale improved outcomes for medical students, particularly males. Educational interventions should be consistent throughout a student's training in any health science discipline^39^. Other educational interventions that have been shown to be effective in training empathy, compassion or communication in students of any health profession are those related to the arts (viewing health-related films, literature or even visualisation of visual art)^36,43,87,88^. More specifically, with physiotherapy students, Chu et al.^89^ report that an empathy training intervention such as video simulation improves students' empathy levels, even more so for females than males.

In terms of gender, future research should consider examining differences in the experiences of men and women during clinical placements: Did men and women have the same opportunities to interact with patients with a wide range of experiences and perspectives? Did they receive feedback on their empathy skills in the same way? This research could help to better understand how clinical practice can contribute to the development of empathy in physiotherapy students and how this impact may be different for men and women.

However, Williams^90^ suggests a shift in how empathy is considered from a self-reported perspective to an emotional communication perspective. He challenges the traditional approach of measuring empathy through self-reporting questionnaires and suggests that empathy should be considered a quality of emotional communication. Building on the said author´s ideas is another consideration to bear in mind for future research with our students as we continue to consider other study tools for empathy.

### Study limitations

The present study may be limited by variability in the professionals who tutored the students and the patients with who the students interacted with. Professionals with different levels of experience, training and teaching techniques supervised the students. Students also interacted with patients with different medical conditions, ages and levels of disability. The variability of professionals and patients could have influenced the study's results. For example, students taught by more experienced professionals or who interacted with patients with more complex medical conditions might have performed differently on the tests.

Another limitation could be the need for comparative studies of assertiveness in health science or physiotherapy students. The existing studies focus on different aspects of assertiveness, use different types of students, and have different results.

The present study was only longitudinal and observational, so it was not possible to establish a cause-and-effect relationship between the studied variables.

## Conclusions

The authors observed that the development of the final year of the physiotherapy degree course, with clinical placement as the primary teaching–learning element, influences the development of empathic thinking in fourth-year physiotherapy students. Although the results are acceptable both before and after clinical placement, there is a decrease in empathic scores, especially in the subscales of perspective taking (PT) and empathic concern (EC) after clinical placement. The erosion of these two empathy subscales is particularly noticeable among male students, especially in the EC subscale.

In the case of assertiveness, the results are also acceptable, although there is an inverse relationship between assertiveness (RAS) and personal distress (PD): the higher the RAS score, the lower the personal distress score. This relationship was maintained in the two evaluations carried out during the course.

As a suggestion for future research, the development of students' empathy and assertiveness during their clinical practice should be further studied, and more longitudinal studies should be proposed, suggesting activities to study and improve these two soft skills.

## Data Availability

The data supporting the findings of the study are available from the corresponding author upon reasonable request. The data are not publicly available due to privacy or ethical restrictions.
